# Oral Health Promotion among Individuals with Intellectual Disabilities: A Systematic Review

**DOI:** 10.1055/s-0043-1768151

**Published:** 2023-05-19

**Authors:** AlBandary Hassan AlJameel, Shabnam Gulzar, Megha Gupta, Alhassan Ali. Alshehri, Sultan A. Almalki, Faris Y. Asiri, Sharmeen J. Chaudhry

**Affiliations:** 1 Department of Periodontics and Community Dentistry, College of Dentistry, King Saud University, Riyadh, Saudi Arabia; 2 Dental Specialist, Pedodontics and Preventive Dentistry, District Hospital Pulwama, Kashmir, India; 3 Department of Pedodontics and Preventive Dentistry, Vyas Dental College and Hospital, Jodhpur, Rajasthan. India; 4 Dentistry Department, North of Riyadh Dental Center, Second Health Cluster, Riyadh region, Ministry of Health, Saudi Arabia; 5 Department of Preventive Dental Sciences, College of Dentistry, Prince Sattam Bin AbdulAziz University, Al-kharj, Saudi Arabia; 6 Department of Preventive Dental Sciences, College of Dentistry, King Faisal University, Al-Ahsa, Saudi Arabia; 7 Independent Researcher, Ohio, United States of America

**Keywords:** dental health, disabilities: education, oral health, promotion

## Abstract

People with disabilities experience inferior health and poor access to good quality health services as compared with the general population. Optimum oral health is associated with improvement in the quality of life in such patients. As oral diseases are largely preventable, good oral health education can have a positive impact on individuals with disabilities. So, the aim of the study was to review the effectiveness of oral health promotion among individuals with intellectual disabilities (IDs). Seven electronic databases were searched using keywords like intellectual disability/mental retardation/learning disability AND dental health education/health promotion. All records that were identified electronically from this search were subjected to a preliminary review to identify eligible papers. Identified studies were grouped into oral health promotion directed at individuals with IDs, and those aimed at caregivers of people with IDs. Interpretation of the outcomes included the effects on oral health knowledge, attitudes, and behaviors (either observed or self-reported). Eventually, 16 studies were included in the review including five studies that were randomized controlled trials, while the remaining 11 studies were pre-post single group oral health promotion studies. Critical appraisal of each study was conducted with the 21-item criteria by Kay and Locker (1997) to provide a numerical quantification and ranking of the evidence. Positive changes in the behaviors and attitudes were observed, while other studies reported a considerable improvement in the knowledge of caregivers for oral healthcare of individuals with IDs. However, such activities need to be done over a long period of time with constant monitoring.

## Introduction

People with disabilities are entitled to good quality of life including their good oral health. However, it has been observed that people with disabilities may experience inferior health and poorer access to good-quality health services as compared with the general population. 1 2 Globally, the burden of oral diseases is large, especially among individuals with disabilities as they already suffer from the impact of the disability itself. 3 The presence of the disability itself increases the individual's expenditure on many sectors such as health, education, and social services. 4 Oral health problems include a range of diseases and conditions that involve dental caries, periodontal diseases, oral cancer, noncarious tooth surface loss, and oral mucosal diseases. 5 Although oral diseases are largely behavioral in origin and notionally preventable, they remain a significant public health challenge in many developed as well as developing countries. 6

Intellectual disability (ID) is a generalized neurodevelopmental disorder characterized by significant impairments in mental capacity and adaptive behaviors (conceptual, social, and practical skills). 7 Individuals with IDs constitute a considerable segment of the population that might increase with time because of the higher disease survival rates after adequate medical care. 8 9 People with IDs, most often, lack the understanding of daily tasks like personal hygiene 6 and proper oral healthcare. 10 Moreover, the barriers to good oral health for individuals with IDs also include low awareness level among caregivers, 11 and their inadequate training. 12 The role of caregivers in providing oral healthcare for individuals they support is very crucial and there is a need to promote regular oral hygiene practices. 13 The ability of self-care and adequate plaque control measures are very crucial elements in maintaining good oral health, as they prevent the occurrence and limit the progression of dental diseases. 14 15 16

Studies have been conducted about health promotion and programs developed for individuals with disabilities. 17 18 19 However, there is a lack of consensus among these studies aimed at evaluating the effectiveness of oral health promotion among individuals with IDs. This is mainly because of the variety of interventions used and the direct target population studied. Therefore, there is a need to compile the findings on such studies and summarize the effectiveness of implemented interventions. Hence, this study aimed to assess the effectiveness of oral health promotion activities/interventions for desired oral health outcomes conducted among individuals with IDs as well as their caregivers by reviewing the available literature.

## Methods

### Search Strategy

Seven electronic databases [Ovid (which includes three databases: Embase, MEDLINE, PsychINFO), Web of Knowledge, CINAHL, The Cochrane Library, and Scopus] were searched using the following keywords: (ID/mental retardation/mental handicap/ learning disability) AND (dental/oral) AND (dental health education/ health promotion/ oral health/ dental hygiene/preventive dentistry/behavior training).

**Fig. 1:**
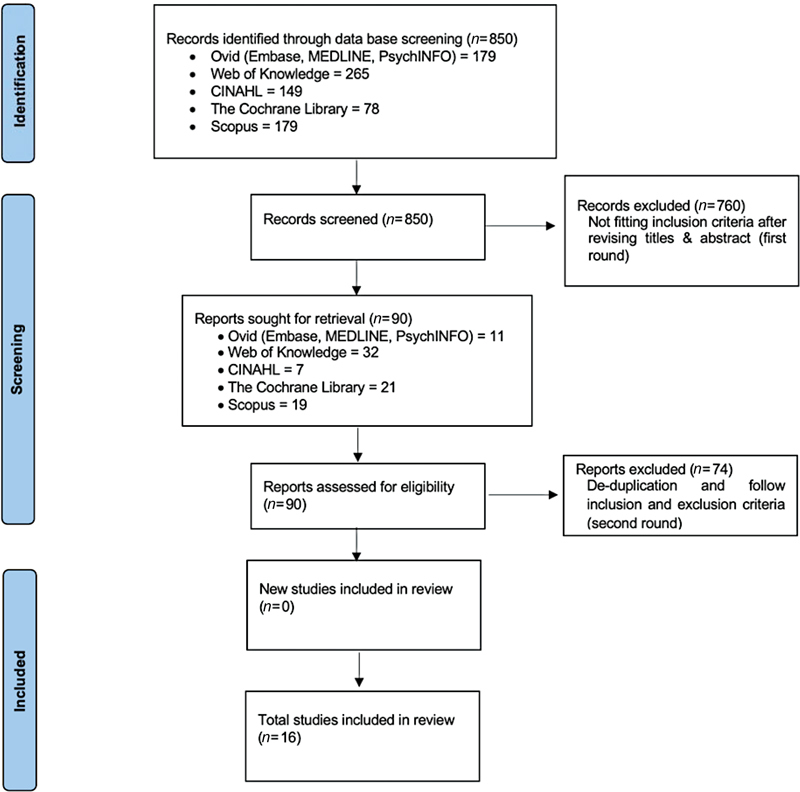
Describes the search strategy according to Preferred Reporting Items for Systematic Reviews and Meta-Analyses (PRISMA) flow diagram.

All records that were electronically identified from this search were subjected to a preliminary review to identify eligible papers by two independent authors. In case of any discrepancy between the two authors, a third author was consulted for general consensus. Reports published more than once from the same study were excluded by reconfirmation of any duplication of reports. The term ID has been previously used as mental retardation or mental handicap. However, such terminology is discriminatory and as such ID is a more accepted term used. 20 The term ID is also used concomitantly with the term “ *learning disability,* ” like in the United Kingdom which sometimes confuses with the term “ *learning difficulty* ”; hence, we preferred to use “ *Intellectual disability* ” consistently for terms learning disability, mental handicap, mental retardation, etc. that have been used historically. Table 1 explains the Population, Intervention, Comparison and Outcome of the referenced studies in accordance with parameters for systematic reviews. 21 The reference list of each retrieved paper was also reviewed. This was done to avoid duplication and to find out any study that might not have been found with the search strategy. An overall 90 papers were retrieved as shown in Fig. 1 that was done according to Preferred Reporting Items for Systematic Reviews and Meta-Analyses (PRISMA) guidelines 2020. 22 Leaving aside individuals with mild IDs, individuals with moderate-to-severe IDs are not able to do their routine oral hygiene procedures by themselves, and it has to be usually done by their caregivers. Therefore, it is important to train the caregivers for proper oral healthcare of individual with IDs. Hence, this study was divided into looking at oral health promotion in individuals with ID and by their caregivers.

**Table 1:** The population, intervention, comparison, and outcome (PICO) parameters for systematic reviews

Parameter	
Population	Individuals with all age-groups classified with intellectual disability or their caregivers
Intervention	Oral health promotion only—like oral health education/oral hygiene instructions Does not include treatment based/pharmacological intervention
Comparison	Not applicable
Outcome	Includes 1. Clinical outcome—plaque levels, oral hygiene status, gingival health 2. Observed /self-reported effects on oral health knowledge, attitude/behavior

Interpretation of the intervention outcomes in this review included both clinical outcomes and the effects on oral health knowledge, attitudes, and behaviors (either observed or self-reported). For each outcome measure, the findings were grouped according to the particular outcome measured, such as plaque, gingival health, oral hygiene as well as knowledge, attitude, and behavior of individuals with IDs. Inclusion/selection criteria has been mentioned in Table 2 .

**Table 2:** Inclusion and exclusion criteria for the studies eventually included in the systematic review

Inclusion criteria	Exclusion criteria
** 1.** Controlled trials (randomized or quasi randomized) that assessed the impact of an intervention/oral health promotion activity on the oral health status of people with intellectual disabilities	1. Studies aimed at treatment-based interventions
** 2.** Manuscripts published in English language only	2. Studies involving professionally applied preventive agents like fluorides, pit and fissure sealants or any antimicrobial agents
** 3.** Papers published in peer-review journals only	
** 4.** All age-groups of above-mentioned criteria were included	

### Focused Research Question

Are oral health promotion activities/interventions either directed at individuals with IDs or at their caregivers effective in improvement of desired oral health outcomes?

### Validity Assessment and Data Extraction

From each study included, data on study details (year study began, place/country where study was conducted, duration of the study), type of study, baseline characteristics of participants (number included, age [mean/range], and settings where participants were recruited), characteristics of the intervention and its follow-up duration, and finally the results of each study were extracted. No date limit was set for the studies. The studies found were classified according to the target of the study population (individuals with IDs and/or their caregivers). A description of each study published in the context of the focused research question that is eventually selected for this review is provided in Tables 3 , 4 , and 5 . Critical appraisal of each study was conducted with the 21-item criteria to provide a numerical quantification and ranking of the evidence as proposed by Kay and Locker in 1997. 23 The quality score was ascertained by calculating the number of affirmative answers to the 21 items. A score of 21 indicates the highest quality of evidence and a score of 0 the weakest quality of evidence, as is represented in Table 3 . However, it should be noted that Kay and Locker assessment criteria was developed mainly to assess traditional randomized control trials, while the majority of retrieved papers were a single-group pre- and post-test studies; therefore, the overall scores of obtained studies were below average. It would be better if more recent/updated checklist criteria are available for quality assessments so that the low scores of quality assessment do not necessarily mean a poor-quality study.

**Table 3:** The quality score of the studies included in the systematic review

Studies quality items	Stefanovska et al 38	Bizarre and Ribeiro 30	Kavvadia et al 34	Shyama et al 37	Altabet et al 24	Shaw and Shaw 25	Reynolds and Block 36	Mac Giolla Phadraig et al 26	Fickert and Ross 32	Glassman and Miller 33	Faulks and Hennequin 31	Lange et al 27	Adiwoso and Pilot 29	Glassman et al 39	Davies and Whittle 28	Nicolaci and Tesini 35
Was the research goal clearly defined?	Yes	Yes	Yes	Yes	Yes	Yes	Yes	Yes	Yes	Yes	Yes	Yes	Yes	Yes	Yes	Yes
Was the intervention fully described for the intervention group?	Yes	Yes	Yes	Yes	Yes	Yes	Yes	Yes	Yes	Yes	Yes	Yes	Yes	Yes	Yes	Yes
Was the intervention fully described for the control group?	NA	NA	NA	NA	Yes	Yes	NA	Yes	NA	NA	NA	Yes	NA	NA	Yes	NA
Was the study population clearly defined?	Yes	Yes	Yes	Yes	Yes	Yes	Yes	Yes	Yes	Yes	Yes	Yes	Yes	Yes	Yes	Yes
Was it stated how subjects were attained?	Yes	Yes	Yes	Yes	Yes	Yes	Yes	Yes	Yes	Yes	Yes	Yes	Yes	Yes	Yes	Yes
Were the subjects clearly defined?	Yes	Yes	Yes	Yes	Yes	Yes	Yes	Yes	Yes	Yes	Yes	Yes	Yes	Yes	Yes	Yes
Was the method of allocation, or similarity between groups described?	NA	NA	NA	NA	Yes	Yes	NA	Yes	NA	NA	NA	Yes	NA	NA	NA	NA
Were groups compared on any variables?	NA	NA	NA	NA	Yes	Yes	NA	Yes	NA	NA	NA	Yes	NA	NA	Yes	NA
Were the outcome measures clearly defined?	Yes	Yes	Yes	Yes	Yes	NA	Yes	Yes	Yes	Yes	Yes	Yes	Yes	Yes	Yes	Yes
Were the outcome measures objectives?	Yes	Yes	Yes	Yes	No	NA	Yes	No	No	Yes	No	Yes	Yes	No	No	Yes
Were the outcome measures tested for validity?	No	No	No	No	No	NA	No	Yes	No	No	No	No	No	No	No	No
Were the outcome measures tested for reliability?	No	No	Yes	Yes	Yes	NA	Yes	No	No	Yes	No	NA	Yes	No	No	Yes
Were the outcome assessors blinded?	No	No	No	No	Yes	NA	No	No	No	No	No	NA	No	No	No	No
Were the participants blinded?	No	No	No	No	No	No	No	No	No	No	No	No	No	No	No	No
Was the statistical analysis appropriate?	Yes	Yes	Yes	Yes	Yes	Yes	Yes	Yes	Yes	Yes	Yes	Yes	Yes	Yes	Yes	Yes
Was the sample size for each group given?	Yes	Yes	Yes	Yes	Yes	No	Yes	Yes	Yes	Yes	Yes	Yes	Yes	Yes	Yes	No
Was there a sample size justification?	No	No	No	No	No	No	No	Yes	No	No	No	NA	No	No	No	No
Was the statistical significance defined?	No	No	Yes	No	No	NA	No	No	No	No	Yes	NA	Yes	No	NA	No
Was the dropout rate given?	No	Yes	No	No	No	No	Yes	Yes	Yes	No	Yes	NA	Yes	No	NA	No
Was the dropout rate <10%?	NA	No	NA	NA	NA	NA	No	No	No	NA	No	NA	Yes	NA	NA	NA
Were the dropouts accounted for?	NA	No	NA	NA	NA	NA	Yes	No	No	NA	No	NA	No	NA	NA	NA
**Quality score**	**9**	**10**	**11**	**10**	**13**	**9**	**12**	**14**	**9**	**10**	**10**	**12**	**13**	**8**	**10**	**9**

Abbreviation: NA, not available.

**Table 4:** General characteristics of studies published on the effects of oral health promotion among individuals with intellectual disabilities (IDs)

Study	Country, year started	Study method	Target population, settings	Intervention	Outcome	Follow-up length	Findings
Stefanovska et al 2010 38	Republic of Macedonia, no date stated	Single intervention group with pre- and post-test	100 schoolchildren with IDs, with age groups 9–13 and 13–16	Educational (supervised tooth-brushing program) to encourage independent manual skills	Oral health index levels (gingival and periodontal health)	6-month intervention program	Program was effective in reducing plaque and gingivitis scores; the key to long-term success is maintenance
Bizarre and Ribeiro 2009 30	Portugal, 2006	Pilot study, single intervention group with pre- and post-test	135 individuals with varying disabilities associated with Intellectual ability enrolled at institution for the disabled, with age range 12–46. Caregivers and parents were also educated on the importance of good oral dietary habits and oral hygiene practices	Educational oral health program (to establish daily tooth-brushing routine)	Intervention outcome was assessed by measuring plaque scores using Simplified Debris Index (DI-S)	Three-month period	Study showed that it is possible to implement daily oral hygiene maintenance in individuals with disabilities, and that most participants adopted more efficient tooth-brushing behavior when monitored daily
Kavvadia et al 2009 34	Greece, no date stated	Single group intervention with pre- and post-test	57 students attending school for young adults with IDs. Mean age: 21 years old	Educational school-based program. Weekly training and supervision of plaque removal for three months	Oral hygiene level (plaque removal efficacy). Using simplified oral hygiene index (OHI-S)	Two years	Program was effective in improving oral hygiene; however, to be effective long-term, it has to be delivered continuously
Shyama et al 2003 37	Kuwait, 2001	Only one group was assessed pre- and post-test	112 children with Down syndrome attending two special needs schools. Age range 11–22	Educational program (supervised tooth-brushing program) and dental health education sessions twice a week	Oral hygiene level (plaque and gingival indexes) PI and GI	Over a period of 3 months	Program was effective in reducing plaque and gingivitis scores; the key to long-term success is sustaining the motivation to make oral hygiene a daily routine
Altabet et al 2003 24	United States of America, 2000	Clinical trial of two intervention groups randomly allocated in the intervention and control group. Oral health examiner was blinded	79 individuals with IDs living at a state residential facility. Intervention = 39 Control = 40	Intervention group received individualized oral care plan. Caregivers of control group were trained on general oral care strategy	Oral hygiene status; level of plaque formation (no standardized measurement of plaque accumulation).	Were examined at two distinct times over a 12-month period (at the beginning and end of the study)	Results showed a modest improvement for all people studied with greater improvement among the treatment group
Shaw an Shaw 1991 25	United Kingdom, no date stated	Randomized controlled trial. Four intervention groups	382 individuals with IDs attending different adult training centers	Dental education G1= baseline OHI & scale and polish. G2= baseline OHI & scale and polish, and daily supervised tooth brushing. G3= baseline OHI & scale and polish, daily supervised tooth brushing, 3-monthly scale and polish. G4= baseline OHI & scale and polish, daily supervised tooth brushing, a monthly scale and polish	Oral hygiene status	Two years	Individuals who were encouraged and motivated were capable of better standards of oral care The input of experienced hygienists on a regular basis improved the periodontal health considerably No clinical significance between one- or 3-monthly professional inputs
Reynolds and Block 1974 36	United States of America, no date stated	Single-intervention group with pre- and post-test.	60 boys at St. Louis School for Exceptional Boys, with age range 8–13 years Only 48 present at initial and final assessment	Oral health educational activity.	Oral hygiene level (plaque scores using PHP index)	Two months	The study showed a dramatic improvement in oral hygiene and concluded that effective tooth brushing could be taught to a group of retarded boys

**Table 5:** General characteristics of studies published on the effects of oral health promotion among caregivers of individuals with intellectual disabilities (IDs)

Study	Country and year started	Study method	Target population and settings	Intervention	Outcome	Follow-up length	Findings
Mac Giolla Phadraig et al 2013 26	Ireland, 2008	Controlled trial with cluster randomization. Response rate = 56.9% with 29.7% attrition	Care staff of community-based residential services Control group = 79 carers. Intervention group = 76 carers.	Educational program (brushing technique and diet table)	Carers' oral health-related knowledge, behavior, attitudes, and self-efficacy	Average time between pre- to post-test questionnaire = 9.5 months (range = 8–11 months)	Mean scores on knowledge, attitude, self-efficacy, and reported behavior significantly increased
Fickert and Ross 2012 32	United States of America, no date stated	One group with pre- and post-test	60 caregivers, 18 years of age or older, working in community living arrangements or intermediate care facility	Educational program (informative presentation, tooth brushing, flossing, and suctioning technique)	Caregivers' oral health-related knowledge, oral skill, and compliance	Outcome measured immediately after intervention and three months later	Significant improvement in knowledge, skill, and compliance in oral hygiene
Glassman and Miller 2006 33	Northern California, no date stated	Single- intervention group; pre and post-test observational study	11 individuals with IDs and 10 caregivers at three community-based residential facilities	Indirect training program; training the caregivers but measuring the ultimate outcome on individuals with ID	Outcome on both caregivers and individuals with ID: 1. Caregivers presence during brushing; 2. Percentage of tooth surfaces brushed; 3. Duration of tooth brushing; and 4. Plaque scores of individuals with IDs	Outcome indicators measured at the baseline and after the completion of the training sessions	Increase in caregiver presence and duration of brushing with concurrent improvement in the oral hygiene of individuals with IDs
Faulks and Hennequin 2000 31	France, no date stated	Single-intervention group; pre- and post-test.	67 residents with IDs (RR = 48%) and 69 caregivers (RR = 62%) at three centers for persons with special needs	Educational program (oral hygiene techniques)	Caregivers' attitudes and behaviors using questionnaire evaluation	At the beginning of the first workshop and then repeated at the last session, between 9 and 12 months later	Improvement in tooth-brushing habits of individuals with IDs; no significant change in caregivers' habits and attitudes toward their own dental health
Lange et al 2000 27	United States of America	Randomized controlled trial	Caregivers at Midwest institution for persons with developmental retardation	Change in policy, staff training on proper tooth-brushing technique, and monitoring by interested third party	Oral hygiene/ plaque index of individuals with IDs	Not stated	Significant improvement in intervention 1 (training and accountability) compared with control and intervention group 2
Adiwoso and Pilot 1999 29	Indonesia. Project started in 1991 and Plaque control program started in 1993	Single-intervention group examined pre- and post-intervention	Intervention on parents of 82 children, teachers and medical staff at rehabilitation institution for handicapped children. Outcome on individuals with IDs	Oral health education program and plaque control program (tooth-brushing and fluoride gel)	Mean plaque scores at the baseline and then seven consequent assessment sessions	Oral health education program performed over 2.5 years	Program was well accepted, effective and of clinical significance
Glassman et al 1994 39	Northern California, United States of America, no date stated	Single-intervention group. Pre- and post-test. Pilot study	20 caregivers (residential care home managers and direct care staff) from six different residential care facilities	Training program (videotape, workbook, instructions for trainers)	Pre and post-test questionnaire	Two training sessions of two hours each. Duration not stated clearly	Participants were able to demonstrate that they could learn the information provided in the educational material and there was improvement in the knowledge level
Davies and Whittle 1990 28	United Kingdom, no date stated	Controlled trial. Intervention = 33 Control = 31	Intervention on 33 caregivers of adults with IDs	Dental health education training sessions (illustrated discussion, tooth-brushing demonstration, practical session)	Effectiveness was assessed using a questionnaire	Seven-week induction course	Significant difference in the knowledge level among intervention and control groups
Nicolaci and Tesini 1982 35	Massachusetts, United States of America 1978	Single-intervention group. Number of trained staff was not stated in the paper	Training the direct care staff to train other direct care staff on proper oral hygiene techniques (to ensure continuous delivery of oral hygiene instructions of adults with IDs)	Training on the oral hygiene program	Effectiveness on the education program was assessed by the oral hygiene index scores (debris score and calculus score) of 84 institutionalized individuals	Study was conducted with an 18-month follow-up period	There was a significant improvement in the oral hygiene index of the examined individuals (a linear trend was shown across the four examination sessions at baseline, 6, 12, and 18 months)

There were two independent authors who read all the articles to find the suitability to be included in the review. A master list for each search engine was made of all retrieved articles and finally selected article list. In case of any discrepancy between the two independent authors, a third author was consulted and after mutual consensus and discussion a decision was arrived at.

## Results

The combined electronic searches revealed 850 papers from the seven databases. Of the 179 papers retrieved, after a preliminary search from Ovid, which include the three databases of Embase, MEDLINE, PsychINFO, only 11 papers were identified as potentially eligible. From the Web of Knowledge search, 265 papers were retrieved, and, using the inclusion criteria, only 32 papers appeared to be potentially relevant. Using CINAHL, 149 papers were retrieved after the preliminary search, and only 7 papers primarily met the inclusion criteria. In the Cochrane Library, the preliminary search revealed 78 (26 Cochrane reviews and 52 Trials), although only 21 seemed to be potentially relevant. And finally, a search on the Scopus database resulted in 179 papers, 20 of which seemed to be of potential relevance to the focused research question. Finally, only 16 studies qualified after application of the selection criteria as mentioned in Table 2 and the study selection process represented in the PRISMA flowchart as Fig. 1 .

Among the 16 included studies, 5 were randomized controlled studies, 24 25 26 27 28 while the remaining 11 studies 29 30 31 32 33 34 35 36 37 38 39 were a pre-/post-single group intervention study as represented in Tables 4 and 5 . Two of the included studies involved a professional input 25 29 however, they were included because they had an educational interventional group as well, so the data on that element only was involved in this study.

Studies were conducted mainly at schools or community-based residential services and nursing homes. The sample size of the target study populations varied considerably from 20 to 382. The follow-up period also varied widely across studies with some having a follow-up period of just a few weeks, while the majority had a follow-up period of 2 years. The results of the search and further discussion is classified based on the target population of the planned intervention. Therefore, studies were grouped into either oral health promotion interventions among individuals with IDs, or oral health promotion interventions among caregivers of individuals with IDs.

### Oral Health Promotion Studies among Individuals with IDs

The search revealed seven studies that aimed to improve the oral health of individuals with IDs by directing their oral health promotion with them directly 24 25 30 34 36 37 38 as is represented in Table 4 . Almost all interventions were educational in nature, and some of them included training programs (e.g., supervised tooth-brushing techniques). Two of them were randomized controlled trials 24 25 and the remaining studies 30 34 36 37 38 were pre- and post-test single-group interventions. In some studies, where the main interventions were directed to individuals with IDs, education of the caregivers was also considered as a part of the intervention. Furthermore, various outcome measures were used to assess the level of dental plaque that made the ability to make comparisons across studies. Several studies compared the effectiveness of powered toothbrushes compared with manual toothbrushes. Comparisons were made of different types of toothbrushes and employed different methods of assessing dental plaque.

### Studies Related to Plaque, Gingival Health, and Oral Hygiene

All included studies, except by Altabet et al 24 assessed the interventions' outcomes by measuring the oral health status of individuals with IDs; however, different measures were used across the studies (e.g., simplified debris index, simplified oral hygiene index, plaque index, or gingival index). The findings of all the intervention studies in general revealed an improvement in the oral hygiene of participants, but the long-term key to success is the maintenance and continuous delivery of such programs.

### Studies Related to Knowledge, Attitudes, and Behaviors

None of the studies considered measuring changes in knowledge, attitudes, and behaviors as a main outcome measure of interventions directed to individuals with IDs, and while some authors reported in their conclusions that their intervention revealed participants' capability of adopting efficient tooth-brushing behavior when monitored daily, they failed to mention exactly how they reached this conclusion.

### Oral Health Promotion Studies among Caregivers of Individuals with IDs

Nine studies 26 27 28 29 31 32 33 35 39 were found in the literature about oral health promotion for caregivers of individuals with IDs as represented in Table 5 . Three of the reported studies were randomized controlled trials, 26 27 28 and the remaining were single groups pre- and post-intervention. All interventions were educational in nature, and were aimed at training the caregivers and improving their knowledge of the proper tooth-brushing and flossing techniques. In Glassman and Miller's 33 study, the researchers tried to implement an indirect training program in which they trained the caregivers and measured the ultimate outcome on the individuals with IDs. Only one study 27 reported a change in the institution's policy that was accompanied by staff training, but no details about the implemented policy were mentioned.

### Studies Related to Plaque, Gingival Health, and Oral Hygiene

Some studies evaluated the effectiveness of the interventions' programs by measuring the oral hygiene of individuals with IDs who were looked after by caregivers involved in the education/training sessions. 27 29 33 35 It was difficult to compare studies because of the different indicators of oral hygiene used (e.g., mean plaque index scores and oral hygiene index); however, all the studies revealed a significant improvement in oral hygiene indicators.

### Studies Related to Knowledge, Attitudes, and Behaviors

Knowledge on oral health among caregivers was assessed using pre- and post-test questionnaires, and then the results were compared. Almost all studies reported a considerable improvement in the knowledge of caregivers after the completion of the intervention period. Changes in the behaviors and attitudes were also reported, with an increase in the number of caregivers during the tooth-brushing session appearing to result in more compliance in oral hygiene. The results also showed that educating and training the caregivers resulted in improvements in the tooth-brushing habits of individuals with IDs.

## Discussion

This study focused on whether oral health promotion interventions among individuals with IDs or their caregivers were effective in desirable oral health outcomes. From the literature search, it was clear that the interventions aimed at improving oral health status among people with IDs can be classified either as directed closely at individuals with IDs or aimed at educating and training the caregivers of people with IDs. Although there are studies available in the literature regarding oral health, access, and interventions among individuals with IDs, 19 40 41 42 not many of these have looked at the effectiveness of such activities. As individuals with ID are just a part of a wide spectrum of disabilities that is growing in number by each day, 43 it has been expressed that there is lack of validated means of measuring the impact of oral health interventions on care providers for individuals with IDs. 31 However, it was observed that the studies included in this review were conducted in a wide variety of settings and of varying study designs. Also, the recruitment criteria of ID participants were inconsistent across the studies. For instance, the participants with all grades of ID (mild/moderate/severe) were included. It is noteworthy that the level of intellectual ability of a person directly effects the means for proper oral hygiene and self-care. In a study, with individuals having varying disabilities ranging from cerebral palsy to ID; Shah et al in 2015 concluded that these individuals have a high unmet oral health need 10 apart from having difficulties of proper oral healthcare access. 41

Individuals with IDs vary in their ability to learn new skills and develop good oral health practices depending on their cognitive ability; however, findings of this study revealed that individuals with IDs who received some training in oral health practices demonstrated their ability to adopt efficient tooth-brushing behaviors when they received systematic instructions, continuous evaluation, and reinforcements. 25 30 36 The majority of studies involved in this review did not assess the cognitive ability of the participants with IDs, so it is not appropriate to generalize the effectiveness of the interventions among all individuals with varying degree of IDs. It is also important to realize that the effectiveness of the implemented interventions was assessed over a relatively short period of time, and therefore long-term effectiveness is still not clear, although most of the researchers stated the need of long-term follow-up and assessment. 11 14 15 Although there is universal acceptance of the fact that more needs to be done in terms of care of individuals with IDs, yet there is a discrepancy in terms of geographical location where certain locations have been found to lack more in terms of oral healthcare of individuals with IDs. 44 In a recent study, it was concluded that longer-term caregiver interventions, as well as on-site support for oral care, are necessary to evaluate outcomes for individuals with developmental and IDs with the goal of reducing the burden of oral disease. However, the same study noted that increased knowledge alone is not adequate to bring about and maintain positive oral health behavior change. Longer-term interventions and more caregiver involvement are important. 45

Most individuals with IDs lack the cognitive ability to complete oral hygiene for themselves, and rely mainly on their caregivers to help them or even to perform it for them. 14 Moreover, caregivers for people with disabilities have been shown to possess a deficiency in adequate oral health attitude. 11 Studies have also shown that caregivers reported a feeling of discomfort or a lack of success when performing oral hygiene practices to their clients (people with IDs) because of inadequate knowledge or training. 11 31 33 It has been recommended that to improve desirable oral health outcomes for caregivers of individuals with disabilities, dental education plus training programs should be prioritized. 11 So, these deficits are considered to be barriers for providing optimal oral care to individuals with IDs, which might risk their health and quality of life in general. 3 41 Although there were some methodological limitations in the studies reviewed, the results of the interventions aimed at improving the oral health knowledge, behaviors, and attitudes of caregivers of individuals with IDs revealed an improvement in the caregivers' oral health knowledge, behaviors, and attitudes as well as the oral hygiene of individuals with IDs. The caregivers may desire more knowledge for the oral healthcare of individuals with IDs. Similarly, parents of individuals with IDs especially during the childhood of individuals with IDs seem to benefit a great deal from oral healthcare advice for their children. After an oral health promotional event in Riyadh, majority of the parents and caregivers of children with special healthcare needs were highly satisfied by oral health education that enabled them to take better care of their children. 46 Therefore, educating and raising awareness about oral healthcare among people who are associated with the care of individuals with IDs seem to be of great importance. Likewise, in a pilot study done by Liu et al in 2021, new approaches like the use of a board game seem to be beneficial for promoting oral health knowledge among individuals with IDs. 47

Focus on the initial and ongoing training of both individuals with IDs as well as their caregivers is very important. 48 A policy system will likely be needed to ensure that consistent and high-quality oral healthcare for people with disabilities in general and with IDs in particular is maintained through broad implementation of healthy lifestyles and desirable oral health behaviors. 24 48 49 50 However, it has also been proposed that a uniform approach to supporting oral health for individuals with developmental and IDs is unlikely to succeed until a system-based approach is adopted to address the diverse needs of such a population. 13 Therefore, future trials are needed that use clearly defined participant characteristics and outcomes to increase precision and decrease bias. Use of validated outcome measures would standardize comparisons used for long-term follow-up.

## Conclusion

The existing studies have shown improvement in desirable oral health outcomes using oral health promotion among individuals with IDs and their caregivers. However, it is difficult to measure the exact effectiveness of such measures unless they are persistent and perseverant in nature. Educational intervention in terms of oral health promotion seems to yield benefit for both individuals with IDs and their caregivers in terms of improved knowledge, attitude, and oral health behaviors, but this needs to be implemented for a long term. More objective measures stating definitive outcomes need to be investigated. Since the effects of such interventions/activities have been slow to meet the needs of this population in general, more research and more such targeted activities are needed.
